# To jump or not to jump: comparing effects of phenotypic plasticity on the visual responses and escape behavior of locusts and grasshoppers

**DOI:** 10.1152/jn.00442.2025

**Published:** 2025-12-09

**Authors:** Soumi Mitra, Saina Namazifard, David Mario Bellini, Alexis Leigh Sarne, Bidisha Halder, Margaret Ruth Eisenbrandt, Aliya Macknojia, Jiayi Luo, Samme Xie, Hojun Song, Chenghang Zong, Fabrizio Gabbiani, Richard Burkett Dewell

**Affiliations:** 1 Department of Neuroscience, Baylor College of Medicine, Houston, Texas, United States;; 2 Quantitative & Computational Biosciences Graduate Program, Baylor College of Medicine, Houston, Texas, United States;; 3 Department of Molecular and Human Genetics, Baylor College of Medicine, Houston, Texas, United States;; 4 Department of Biology, Emory University, Atlanta, Georgia, United States;; 5 Department of Entomology, Texas A&M University, College Station, Texas, United States

**Keywords:** DCMD, LGMD, locust, looming, phenotypic plasticity

## Abstract

Locusts exhibit remarkable phenotypic plasticity, changing their appearance and behavior from solitary to gregarious when population density increases. These changes include morphological differences in the size and shape of brain regions, but little is known about plasticity within individual neurons and alterations in behavior not directly related to aggregation or swarming. We investigated looming escape behavior and the properties of a well-studied collision-detection neuron in gregarious and solitarious animals of three closely related species, the desert locust (*Schistocerca gregaria*), the Central American locust (*S. piceifrons*), and the American bird grasshopper (*S. americana*). For this neuron, the lobula giant movement detector (LGMD), we examined dendritic morphology, membrane properties, gene expression, and looming responses. This is the first study done on three different species of grasshoppers to observe the effects of phenotypic plasticity on the jump escape behavior, physiology, and transcriptomics of these animals. Unexpectedly, there were few differences in these properties between the two phases, except for behavior. For the three species, gregarious animals jumped more than solitarious animals, but no significant differences were found between the two phases of animals in the electrophysiological and transcriptomic studies of the LGMD. Our results suggest that phase change impacts mainly the motor system and that the physiological properties of motor neurons need to be characterized to fully understand the variation in jump escape behavior across phases.

## NEW & NOTEWORTHY

Some grasshopper species swarm, called locusts. We compared jump escape behavior between gregarious and solitarious grasshoppers and locusts, as well as LGMD responses to looming stimuli, and analyzed potential physiological differences in this sensory neuron. This study provides insights into the effects of phase change on the visual system of locusts and grasshoppers as it relates to looming-evoked jump escape behavior. In this context, our results suggest that phenotypic plasticity mainly impacts the motor system.

## INTRODUCTION

Phenotypic plasticity is defined as an organism’s ability to change its phenotype in response to environmental changes. The plasticity could be behavioral, morphological, developmental, physiological, or reproductive (1–4). Desert locusts (*Schistocerca gregaria*) offer a remarkable example of phenotypic plasticity. In response to an increase in population density, cryptic solitarious animals turn into gregarious locusts of conspicuous color. The bidirectional, density-dependent switch between the solitarious and gregarious phases of locusts is known as phase polyphenism. This property has led to locusts being one of the most extensively studied animal models of phenotypic plasticity (2, 5–8). Plastic phenotypic changes are expected to be beneficial. For instance, a change in color helps solitarious locusts merge with their surroundings to avoid predation (9, 10). In dry climatic regions, when food sources are plentiful after rainfall, the population of initially rare locusts increases until it reaches a point where vegetation becomes scarce due to foraging. This often leads to solitarious locusts (usually at densities of <3 animals per 100 m^2^) encountering each other on resource patches, thus triggering aggregation (reaching 100,000 animals per 100 m^2^). Solitarious individuals show significant gregarious characteristics within 4–8 h of aggregation (2). The degree of phase polyphenism in grasshoppers of the *Schistocerca* genus differs between species, with some exhibiting pronounced changes, for example, the desert locust (*S. gregaria*) and the Central American locust (*S. piceifrons*); no phase change, for example, the gray bird grasshopper (*S. nitens*); or intermediate polyphenism, for example, the American bird grasshopper (*S. americana*; 3, 6, 11).

Like other sighted animals, locusts and grasshoppers rely on vision to avoid collision or predation. Looming sensitive neurons that respond selectively to objects approaching on a collision trajectory have been described in several species, including pigeons (12, 13), vinegar flies (*Drosophila melanogaster* (14–18), zebrafish (19–22), and grasshoppers (23–26). Postsynaptic to the lobula giant movement detector (LGMD), the descending contralateral movement detector (DCMD) neuron in grasshoppers is involved in triggering escape behavior (27, 28). The LGMD is in the optic lobe and synapses with the DCMD, resulting in 1:1 spiking. The DCMD axon faithfully relays spike trains from the brain to the metathoracic ganglion, where it synapses with the motoneurons that play a key role in jumping (27, 29). Electrophysiological studies and modeling of the LGMD have produced detailed descriptions of its active membrane properties and their roles in neuronal computation (30–34).

**Figure 1.**
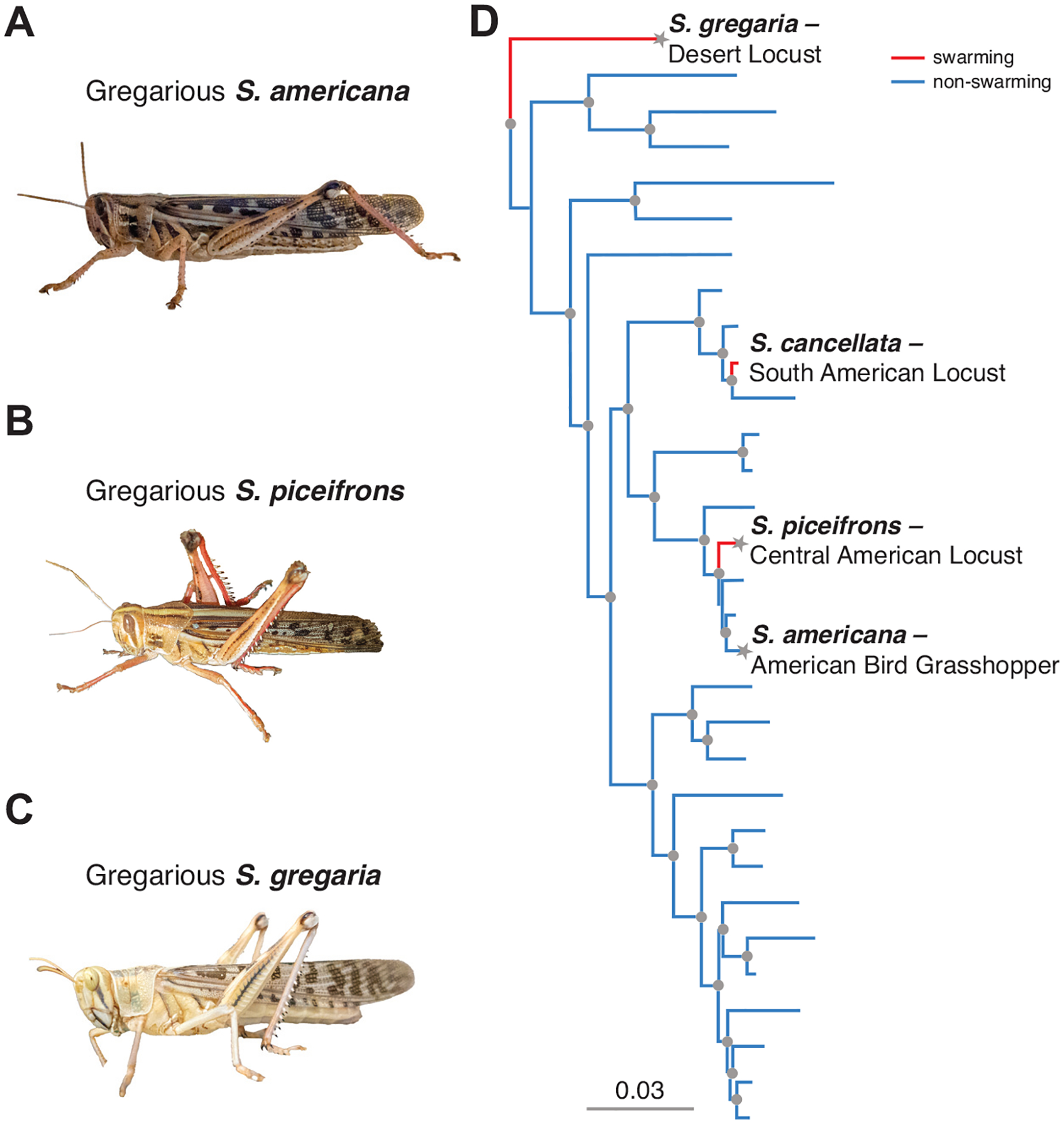
*A*–*C*: visible differences in coloration of the adults within the gregarious (gregarious phase) animals belonging to *Schistocerca americana*, *S. piceifrons*, and *S. gregaria*. *D*: Bayesian phylogenetic tree showing that *S. piceifrons* (swarming species) and *S. americana* (nonswarming species) emerged from a lineage that shares a common ancestor with swarming *S. gregaria* (studied species indicated by stars; scale bar indicating evolutionary distance reports expected sequence substitution rate). The red bars represent swarming species and illustrate that swarming has independently evolved multiple times in the *Schistocerca* genus, including in the South American Locust (*S. cancel-lata*). *D* adapted from Song et al. (3). Reprinted with permission under Creative Commons Attribution 4.0 International License.

Previous research on the effect of phase change in desert locusts found gross anatomical density-dependent differences in the brain and optic lobe morphologies (35, 36). In addition, DCMD responses to looming stimuli were reported to be typically higher in gregarious desert locusts than solitarious ones (37, 38). Whether these phase differences result in differences for escape behavior has not been tested. We thus assessed the impact of density-dependent phase change on jump escape behavior and LGMD properties in species with varying degrees of polyphenism (*S. gregaria, S. piceifrons*, and *S. americana*; Fig. 1).

Here we demonstrate a dramatic density-dependent plasticity in escape behavior, wherein gregarious animals of each species jumped more frequently than the solitarious ones. We then conducted a detailed study of the electrophysiological, anatomical, and transcriptional characteristics of the LGMD neuron within both phases of *S. gregaria* and *S. americana* in pursuit of the underlying causes of the behavioral difference. Unlike previous investigations of *S. gregaria* (37, 38), we did not find substantial differences in the DCMD firing between the two phases of either species. Furthermore, there were no differences in the LGMD dendritic morphology, membrane properties, or gene expression between phases that could account for the behavioral differences. These data suggest that the phase-dependent difference in escape behavior is due to phenotypic plasticity of motor output rather than sensory processing.

## MATERIALS AND METHODS

### Animals

All experiments were conducted using 8–12 wk old adults of both male and female grasshoppers (*S. americana*) and locusts (*S. gregaria* and *S. piceifrons*). Gregarious animals were reared in a dense colony, and solitarious animals were reared individually in small cages, preventing them from touching, seeing, or smelling each other (5, 6). Both groups were reared under 12:12-h light-dark conditions and had been in solitarious or gregarious conditions for at least two generations (39). Animals were selected for health and size without randomization and investigators were not blinded to experimental conditions. Due to space constraints, species availability related to the seasonality of field expeditions, and legal restrictions on the use and maintenance of non-native locust species in the United States, we had to limit the number of experimental animals as described in Electrophysiological Experiments and Staining under LGMD Staining and Imaging. However, the number of replicates tested in this study is sufficient to draw adequate statistical conclusions.

### Visual Stimuli

Visual stimuli were generated using MATLAB (RRID: SCR_001622; see https://scicrunch.org/resources for details) and the Psychophysics Toolbox (PTB-3, RRID:SCR_002881) on a personal computer. For DCMD experiments, stimuli were presented on a cathode ray tube monitor refreshed at 200 frame/s (LG Electronics, Seoul, Korea). For behavior experiments, a monitor operating at 240 frame/s was used (Acer Predator XB253Q). Looming stimuli are the two-dimensional (2-D) projections of an object approaching on a collision course with the animal at constant velocity. Two different types of looming stimuli were used for extracellular DCMD recordings: black (OFF) and white (ON) presented on a background of opposite polarity. For jump experiments, only black stimuli were used. Looming stimuli comprised a square simulating a solid object with half-size *l* approaching at constant speed *v* and were shown at the screen’s maximum contrast. As previously described, the expansion profile is characterized by the ratio *l*/|*v*| (in units of time; 23, 32). Repeated stimuli with different *l*/|*v*| values were presented in pseudo-random order (30).

### Jump Experiments

Jump experiments were conducted at three different *l*/|*v*| values: 20, 50, and 80 ms. These values were previously found to elicit reliable escape behavior (31, 40, 41). Adult grasshoppers of both sexes were used for these experiments. Stimuli were shown to the animals with at least a 10-min interval between two consecutive trials. A total of 10 gregarious *S. piceifrons*, 9 solitarious *S. piceifrons*, 10 gregarious *S. gregaria*, 10 solitarious *S. gregaria*, 15 gregarious *S. americana*, and 21 solitarious *S. americana* were used. Each animal was shown up to 50 stimuli over up to 2 wk of testing. Trials where the animal moved on the testing platform and did not align with the stimulus were excluded from analysis. Videos were recorded at 200 frames/s with a high-speed digital CMOS camera (BFS-U3–16S2M, FLIR), equipped with a 12 mm lens (MVL12M23; Navitar). We synchronized the stimulus and video using TTL pulses transmitted to the camera from the computer, generating the visual stimulus via a universal serial bus (USB) to transistor-transistor logic (TTL) cable (FTDI TTL-232R-5V-WE). Videos were made from 8-bit images and saved in lossless motion JPEG format using custom MATLAB code. The time when the animal jumped relative to the collision was calculated based on the synchronization of the camera recording with the stimulus onset.

### Electrophysiological Experiments

The surgical procedures used in these experiments have been previously described (31, 42, 43). For extracellular DCMD recordings, an adult animal was mounted ventral side up on a narrow plastic holder. The cuticle around the neck area was carefully removed with the help of forceps to expose the nerve cords. The animal was then secured on a stand in front of the monitor and a pair of Formvar-coated stainless steel wire hooks was placed on the ventral nerve cord between the suboesophageal and prothoracic ganglia to record extracellular DCMD responses to visual stimuli (32). Earlier studies showed that intertrial interval duration affects DCMD responses through habituation (44, 45). A 2-min delay between consecutive trials supplemented with mechanical stimulation by brushing the tail, jingling keys, and turning the room lights on and off multiple times was used to minimize DCMD habituation (40). During experiments, room-temperature saline was used to retain moisture around the nerve cords. Ten repetitions of stimuli with *l*/|*v*| values equal to 20, 40, 60, and 80 ms were shown to all *S. gregaria*; 10 repetitions of stimuli of *l*/|*v*| values equal to 20, 50, and 80 ms were shown to all *S. americana*. In total, 8 solitarious *S. gregaria*, 9 gregarious *S. gregaria*, 9 solitarious *S. americana*, and 10 gregarious *S. americana* were used. For this part of the study, we did not use *S. piceifrons* due to its unavailability at the time these experiments were conducted.

Intracellular LGMD recordings were done in discontinuous current-clamp mode using thin-walled borosilicate glass pipettes filled with a solution containing 1.0 M potassium acetate and 1.5 M KCl, yielding electrode resistances of 12–20 MΩ (pipette outer/inner diameter: 1.2/0.9 mm; WPI, Sarasota, FL; 31, 43). The LGMD synapses with the DCMD, which leads to a unique 1:1 spike pattern. This characteristic was used to identify the LGMD accurately while the spikes from DCMD were recorded extracellularly with hook electrodes placed on the ventral nerve cord. We targeted the base of dendritic *field A* based on its location relative to the tracheas of the respiratory system that act as reliable anatomical landmarks. An additional criterion used was the height of intracellular spikes, which typically ranges from 25 to 40 mV at the base of *field A* (46). Step currents of ±1, ±2, and ±5 nA were used. Membrane potential (*V*_m_) and current (*I*_m_) were low-pass filtered with cut-off frequencies of 10 kHz and 5 kHz, respectively. Recordings were digitized at a sampling rate of at least 20 kHz. We used a single-electrode amplifier capable of operating in discontinuous current-clamp mode (typically 3 kHz; Axoclamp 2B, Molecular Devices). For these experiments, four solitarious, five gregarious *S. americana*, and three gregarious *S. gregaria* were used.

### LGMD Staining and Imaging

#### Animal preparation.

A mature locust was mounted on a plastic holder, dorsal side up, and immobilized by vacuum grease. Except for the right eye used for visual stimulation, the head and neck were bathed in ice-cold locust saline (47). The cuticle on the head between the two eyes was partially removed to expose the head capsule, where the two optic lobes and the brain are located. The gut was completely removed. The muscles in the head capsule were also removed. The cuticle around the neck was removed, leaving only two pairs of trachea and the nerve cords. The right optic lobe was desheathed with the help of fine forceps (Dumont No. 5; Dumont SA, Switzerland). A metal holder was inserted underneath the right optic lobe to elevate and stabilize it during intracellular recordings (42).

#### Staining.

A sharp intracellular electrode (thin-walled borosilicate glass pipette, see above) was filled with ~3 μL of a solution of 1.0 M potassium acetate, 1.5 M KCl, and 0.9 μL of 10 mM Alexa Fluor 594, hydrazide at its tip (Thermo Fisher Scientific, Waltham, MA). The dye was injected into the LGMD for a few minutes by iontophoresis using a small negative current passing through the electrode (−1 nA). 13 solitarious *S. gregaria*, 17 gregarious *S. gregaria*, and 8 gregarious *S. americana* were used. At the time these experiments were conducted, isolated *S. americana* were not available.

#### LGMD dendritic morphology.

For dendritic reconstructions, stained LGMDs were imaged using a custom-built two-photon excited fluorescence laser scanning microscope (48). Two-photon image stacks of the LGMD were acquired using ScanImage (RRID:SCR_014307) and processed using MATLAB for further analysis. The LGMD image files obtained post processing were then imported into neuTube software (49) for tracing, followed by Sholl morphological analysis using the TREES Toolbox (RRID:SCR_010457; 50). Next, compartmental modeling of passive properties was performed using NEURON software (RRID:SCR_005393), as described in Ref. 34.

### LGMD passive simulations.

To estimate the unknown parameters *R*_m_, *R*_a_, and *C*_m_ by fitting current clamp data, the LGMD morphology was implemented in NEURON and used to simulate current clamp experiments in a passive model. The time constant τ_m_ was obtained by fitting the initial exponential portion of the experimental membrane potential response. *R*_m_ and *R*_a_ were estimated by fitting the passive steady-state model output to the experimental data, using the least-squares method. Both *R*_a_ and *R*_m_ were assigned uniformly across all the segments in the NEURON model. *C*_m_ was calculated from the relation *C*_m_ = τ_m_/*R*_m_.

### Single-cell RNA-sequencing of LGMD soma.

After intracellular staining with Alexa 594 fluorophore as described in Staining above, the cell bodies of the LGMD were extracted from the lobula using a suction pipette. The rest of the dissected lobula and LGMD cell bodies were stored in Trizol at −80°C. cDNA was obtained followed by single-cell RNA-Seq of the genes expressed in the LGMD and the lobula using snapTotal-seq (51). Twenty-six solitarious *S. gregaria* animals were used, with 12 cell bodies successfully extracted, and 8 yielded RNA sequencing data passing quality control tests. For gregarious *S. gregaria*, 28 animals yielded 11 somas and 7 yielded usable RNA sequencing data.

After sequencing, data were mapped to the *Schistocerca gregaria* genome using the STAR aligner (52). Reads with duplicate unique molecular identifiers (UMIs) were filtered out and the count matrix was generated using UMI tools (53). Then, the data were analyzed using edgeR, an R package for RNA-Seq analysis (RRID:SCR_012802; 54). The genes were tested for significant differential expression using the quasi-likelihood *F* test, and then the *P* values were corrected for multiple testing using the Benjamini-Hochberg method.

### Data analysis and statistics.

Data was analyzed using custom MATLAB code (The MathWorks, Natick, MA). Fisher’s exact test was performed to look for statistical significance in jump frequency, whereas the Wilcoxon Rank Sum test was used to determine statistical significance of differences in the jump time values before collision. Jump probabilities were calculated based on the median unbiased estimator of a binomial response model. Error bars shown in the results are 95% confidence intervals of the probability estimates. Estimates of the DCMD instantaneous firing rate (IFR) were computed by convolving individual spike trains with a Gaussian function (standard deviation, SD: 20 ms) as described earlier (23). Individual means of IFR and peak time relative to collision to each stimulus for each animal were calculated first, followed by population means to each stimulus across animals in each group. An ANCOVA test was done to check for any statistically significant difference in the mean time and amplitude of the peak instantaneous firing rate. For the input resistances in Fig. 5, we measured the change in median membrane potential during the 0.5 s before current onset and the median potential 0.015–0.25 s after onset. The resulting change in potential was divided by the corresponding membrane current amplitude to calculate the input resistance for that step. A Kruskal–Wallis test was performed to check for significant differences in membrane input resistances.

## RESULTS

Here, we studied differences in escape behavior between long-term gregarious and solitarious phases of three different species of locusts and grasshoppers: *S. piceifrons*, *S. gregaria*, and *S. americana*. *S. gregaria* and *S. piceifrons* are bona fide locust species, whereas *S. americana* exhibits an intermediate phenotype with some characteristics of swarming species, despite the fact that it is a grasshopper and does not swarm in its natural environment (11).

### Gregarious Animals Jumped More Than Solitarious Ones

**Figure 2.**
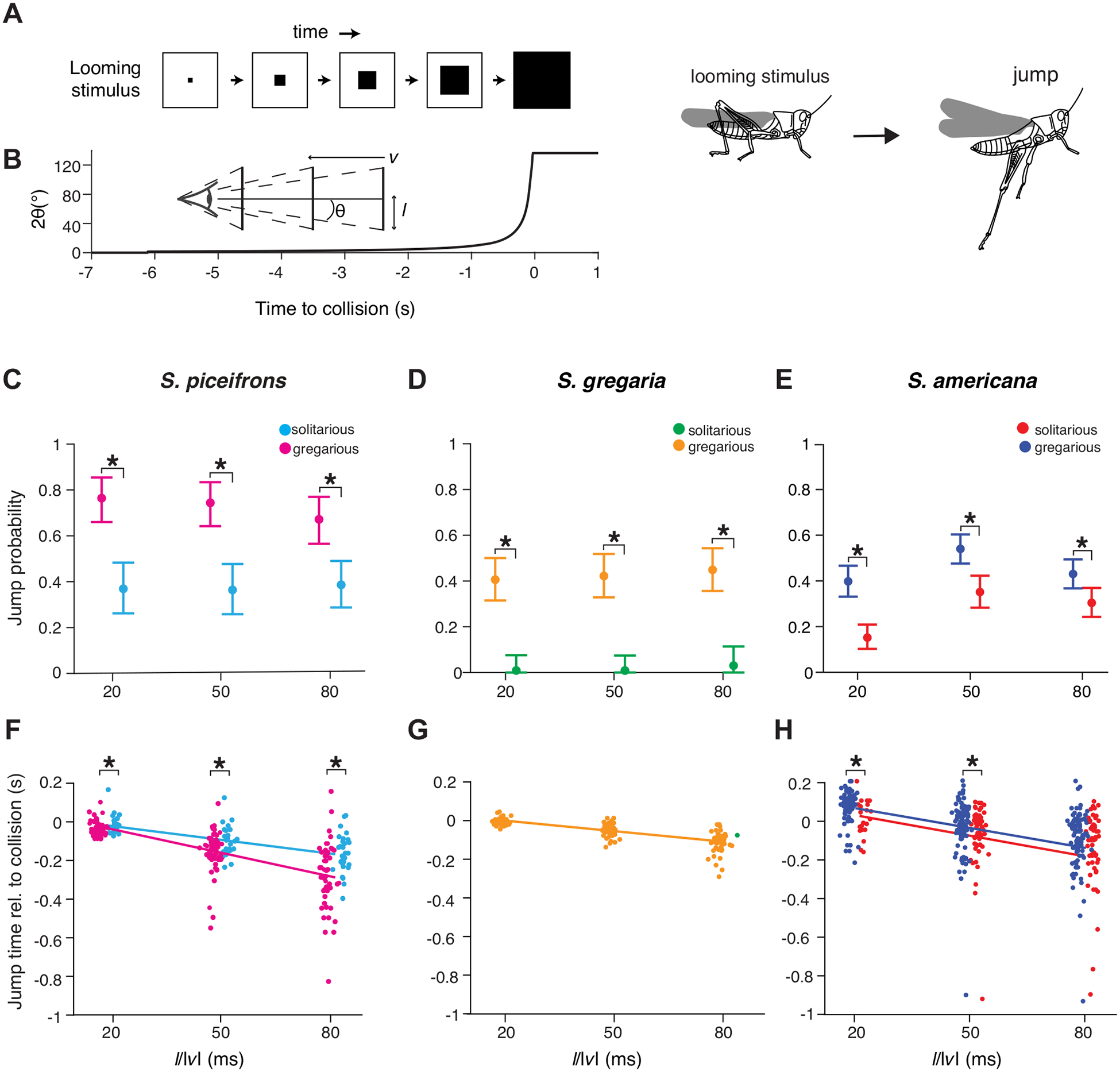
Jump probabilities were lower for solitarious locusts and grasshoppers. *A*: schematic illustration of looming stimulus. *B*: time course of loom’s angular size illustrating its nonlinear progression during a jump experiment (*l*/|*v*|=80 ms; *l* is half length and *v* is velocity). *C*–*E*: jump probability of locusts (*S. piceifrons*, solitarious *n* = 9, gregarious *n* = 10; *S. gregaria*, solitarious *n* = 10, gregarious *n* = 10) and grasshoppers (*S. americana*, solitarious *n* = 21, gregarious *n* = 15). Gregarious animals jumped more in response to looming stimuli. Error bars are 95% confidence intervals of the probability estimates. *F*–*H*: linear fit of jump times before collision. *P < 0.05, Fisher’s exact test (*C*–*E*) and Wilcoxon rank-sum test (*F* and *H*).

Solitarious animals had been reared separately, which prevented them from touching, seeing, or smelling conspecifics for at least two generations. Gregarious animals were reared for multiple generations in a high-density laboratory colony. For each behavioral trial, an unrestrained animal was shown a black looming stimulus once it walked on a platform and its eye was aligned with the center of the stimulus (Fig. 2A). Three different *l*/|*v*| values were used for this experiment where *l* is the half-length of the simulated approaching square, *v* is the approach velocity, and θ is the angular half-size of the looming stimulus subtended at the retina (Fig. 2B). Gregarious *S. piceifrons* jumped more for all stimuli than solitarious *S. piceifrons* (Fig. 2C; *P*_FE_ = 1.9·10^−6^, 3.2·10^−6^, 0.0003; Fisher’s exact test). Similarly, jump probabilities for gregarious *S. gregaria* at the three *l*/|*v*| values were higher than those of the solitarious *S. gregaria* (Fig. 2D). Only one of the ten tested solitarious *S. gregaria* jumped during a loom—in response to one stimulus with *l*/|*v*| of 80 ms; none of the other nine solitarious *S. gregaria* animals jumped during a loom resulting in different jump probabilities for solitarious versus gregarious animals (Fig. 2D; *P*_FE_ = 8.16·10^−8^, 2.92·10^−8^, 2.27·10^−7^). For *S. americana*, the jump probabilities were also higher for gregarious animals at all three *l*/|*v*| values (Fig. 2E; *P*_FE_ = 1.1·10^−7^, 0.002, 0.0071). We also compared the timing of escape jumps relative to the time of projected collision. Gregarious *S. piceifrons* jumped earlier than the solitarious ones for each stimulus (Fig. 2F; *P*_WRS_ = 0.034, 0.044, 0.007; Wilcoxon Rank sum test). Due to the lack of jumps from solitarious *S. gregaria* (Fig. 2G), no statistical comparison could be made for their jump timing. Gregarious *S. americana* jumped later than solitarious ones for the two faster looming stimuli (Fig. 2H; *P*_WRS_ = 0.0039, 0.046, 0.146). In summary, long-term gregarious animals jumped more than solitarious animals for every stimulus tested, but no consistent differences in jump timing were found across species.

### Neural Activity Did Not Differ between Phases

**Figure 3.**
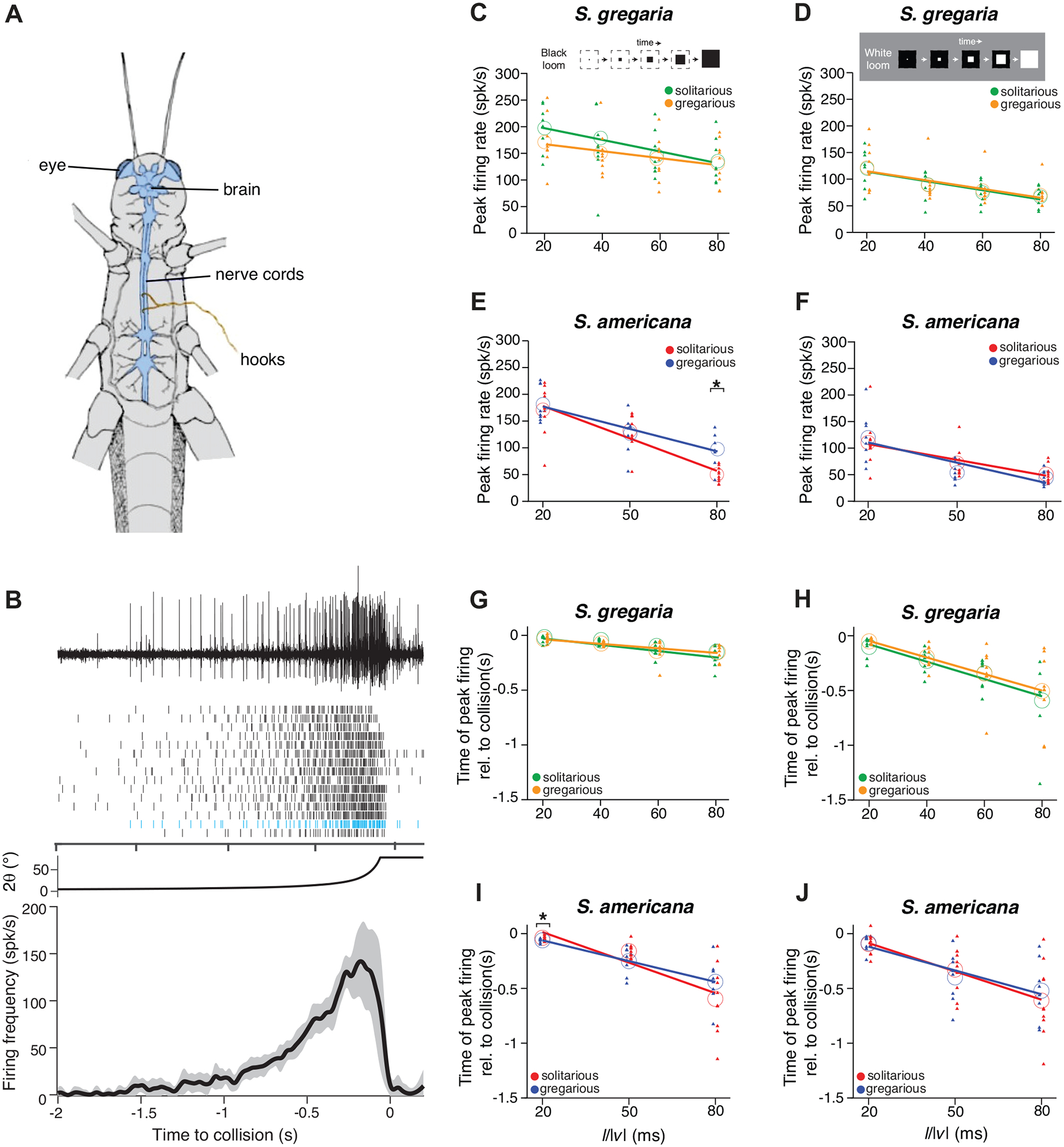
Comparison of DCMD responses between phases of locusts and grasshoppers. *A*: illustration of DCMD recording with wire hooks on the grasshopper’s ventral nerve cord. *B*: example recording and spike rasters of DCMD (*top*) from a solitarious *S. americana* animal (raster of example trace indicated in cyan), stimulus angular size (*middle*) and means ± SD of DCMD instantaneous firing rate (IFR; *bottom*) for an example animal in response to looming stimuli with *l*/|*v*| = 50 ms. *C* and *D*: peak IFR to black and white looms in solitarious and gregarious *S. gregaria* at four *l*/|*v*| values. Triangles show individual means; empty circles show population means and lines represent linear fit of individual means. *E* and *F*: peak IFR to black and white looms in solitarious and gregarious *S. americana* at three different *l*/|*v*| values, plotted as in *C* and *D*. Gregarious *S. americana* had higher firing rates than solitarious animals for *l*/|*v*| = 80 ms. *G* and *H*: time of the peak firing relative to collision for black (*G*) and white (*H*) looms in solitarious and gregarious *S. gregaria* at four *l*/|*v*| values. *I* and *J*: peak IFR timing of *S. americana*, plotted as in *G* and *H*. Gregarious *S. americana* had earlier responses for black looms with *l*/|*v*| = 20 ms. *P < 0.05, Wilcoxon rank sum test. Solitarious *S. gregaria n* = 8, gregarious *n* = 9; solitarious *S. americana n* = 10, gregarious *n* = 8. DCMD, descending contralateral movement detector.

Looming-evoked jumps are triggered by descending activity of the DCMD neuron (28, 30, 31), so we examined DCMD looming responses in the two phases of *S. gregaria* and *S. americana*. DCMD activity was recorded by placing a pair of wire hooks on the nerve cord (Fig. 3A). White and black looming stimuli of multiple half-size to speed ratios were presented to the contralateral eye, DCMD spikes were identified by spike thresholding, and instantaneous firing rates (IFRs) were calculated (Fig. 3B). The peak IFR in response to black and white looms were measured (Fig. 3, C–F). Only the *S. americana* responses to black looms with *l*/|*v*| of 80 ms were different between phases, with gregarious animals showing higher firing than solitarious ones (Fig. 3E; *P*_WRS_ = 0.002, Wilcoxon rank sum test).

We also compared the response timing for each stimulus (Fig. 3, G–J). No differences were found in the time of peak firing between gregarious and solitarious *S. gregaria* (Fig. 3, G and H). For *S. americana*, the peak time of black loom responses was earlier for gregarious animals (Fig. 3I; *P*_WRS_ = 0.043, Wilcoxon rank sum test). The response timing to white looms did not differ for solitarious and gregarious *S. americana* (Fig. 3J; *P*_WRS_ = 0.9654, 0.514, 0.633, Wilcoxon rank sum test). Overall, our data showed that there was no consistent statistical difference between the DCMD responses in either species. No comparisons were made across species.

### LGMD Morphology Is the Same in Solitarious and Gregarious Animals

**Figure 4.**
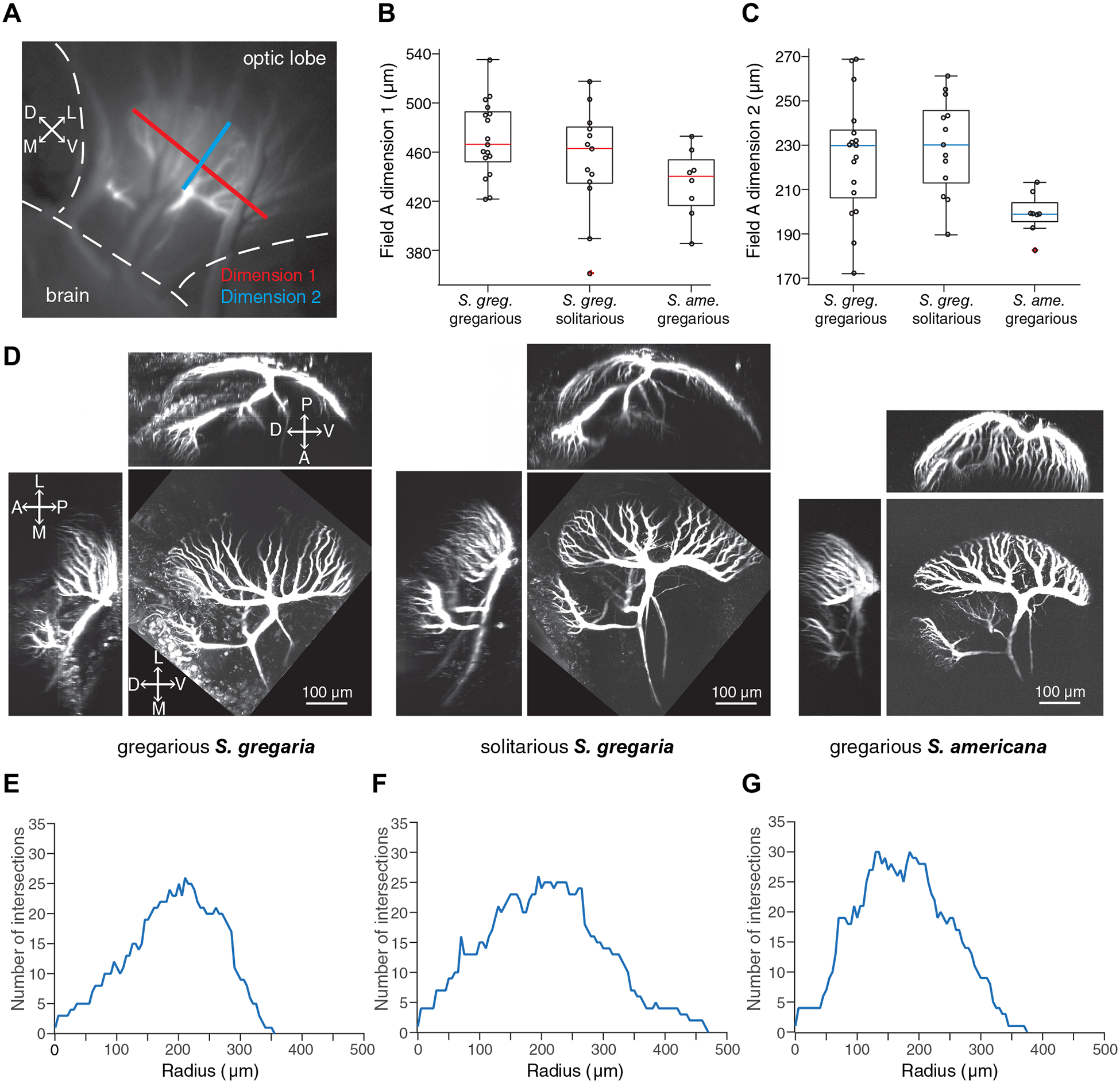
LGMD morphology across phases and species. *A*: charge-coupled device (CCD) image of an LGMD stained with Alexa 594. The size of the dendritic *field A* was measured in two different dimensions: approximately dorso-ventral (dimension 1, red) and approximately medial-lateral (dimension 2, blue). *B* and *C*: for both dimensions, *S. americana* LGMD was smaller (*P* = 0.040 and *P* = 0.002; Wilcoxon rank sum test), but there were no significant differences in LGMD size between gregarious and solitarious *S. gregaria* (*P* = 0.40 and *P* = 0.56). Empty black circles represent individual data points (13 solitarious *S. gregaria*, 17 gregarious *S. gregaria*, 8 gregarious *S. americana*). *D*: two-photon stacks of the LGMD showing smaller LGMD in *S. americana* (*right*). *Insets* in *left* images show the approximate anatomical coordinates. Image courtesy: Dewell and Gabbiani (31). Reprinted with permission under Creative Commons Attribution 4.0 International License. *E–G*: Sholl analysis of LGMD of gregarious *S. gregaria*, solitarious *S. gregaria*, and gregarious *S. americana* showed that the distribution of intersections was significantly different in gregarious *S. americana* than gregarious *S. gregaria* (*P* = 0.0006) and solitarious *S. gregaria* (*P* = 0.0002), but not between phases in *S. gregaria* (*P* = 0.50; Kolmogorov–Smirnov test). A, anterior; D, dorsal; L, lateral; LGMD, lobula giant movement detector; M, medial; P, posterior; V, ventral.

So far, we observed that gregarious animals jumped more than the solitarious ones, but DCMD activity was similar between the two phases. Gross anatomy of the brains and optic lobes are different between phases of *S. gregaria* (36), but individual neuron morphology has not been compared. We examined whether phenotypic plasticity has any significant effect on the morphology of the LGMD. To answer this question, we stained the LGMD by intracellular injection of fluorescent dye in both phases of *S. gregaria* and gregarious *S. americana* (Fig. 4). The LGMD has three distinct dendritic fields with the largest, *field A*, receiving retinotopically mapped excitation. We examined the size and branch morphology of dendritic *field A*, as it is the field most consistent in size and shape across animals and also the most critical region for dendritic processing (30, 31, 55). For the dorso-ventral measurements, the median size of field A in *S. gregaria* was ~460 μm for both phases (*P*_WRS_ = 0.40), whereas that of gregarious *S. americana* was smaller (~440 μm; *P*_WRS_ = 0.040; Fig. 4B). Similarly, the median size of *field A* when measured medial-laterally in gregarious and solitarious *S. gregaria* was ~230 μm (*P*_WRS_ = 0.56), the median size of *field A* in gregarious *S. Americana* was ~200 μm, smaller than *S. gregaria* (*P*_WRS_ = 0.002; Fig. 4C). The 3-axis maximum projections of two-photon stacks for selected example LGMDs show that the LGMD in *S. americana* is slightly more compact, with similar morphology as those of *S. gregaria* across phases (Fig. 4D). To further characterize the LGMD morphology, we performed Sholl analysis on the dendritic branching within *field A* (56). This analysis examines the number of dendritic branches within each radial distance from the base of the dendritic field (Fig. 4E). Like with the 2-D measures (Fig. 4, B and C), the Scholl analysis showed no difference in dendritic morphology between phases of *S. gregaria* (*P*_KS_ = 0.50), but that the gregarious *S. americana* LGMD was more compact than either (Fig. 4, E–G; *P*_KS_ = 0.0006, 0.0002, Kolmogorov–Smirnov test).

### LGMD Input Resistance Was Consistent between the Two Phases

**Figure 5.**
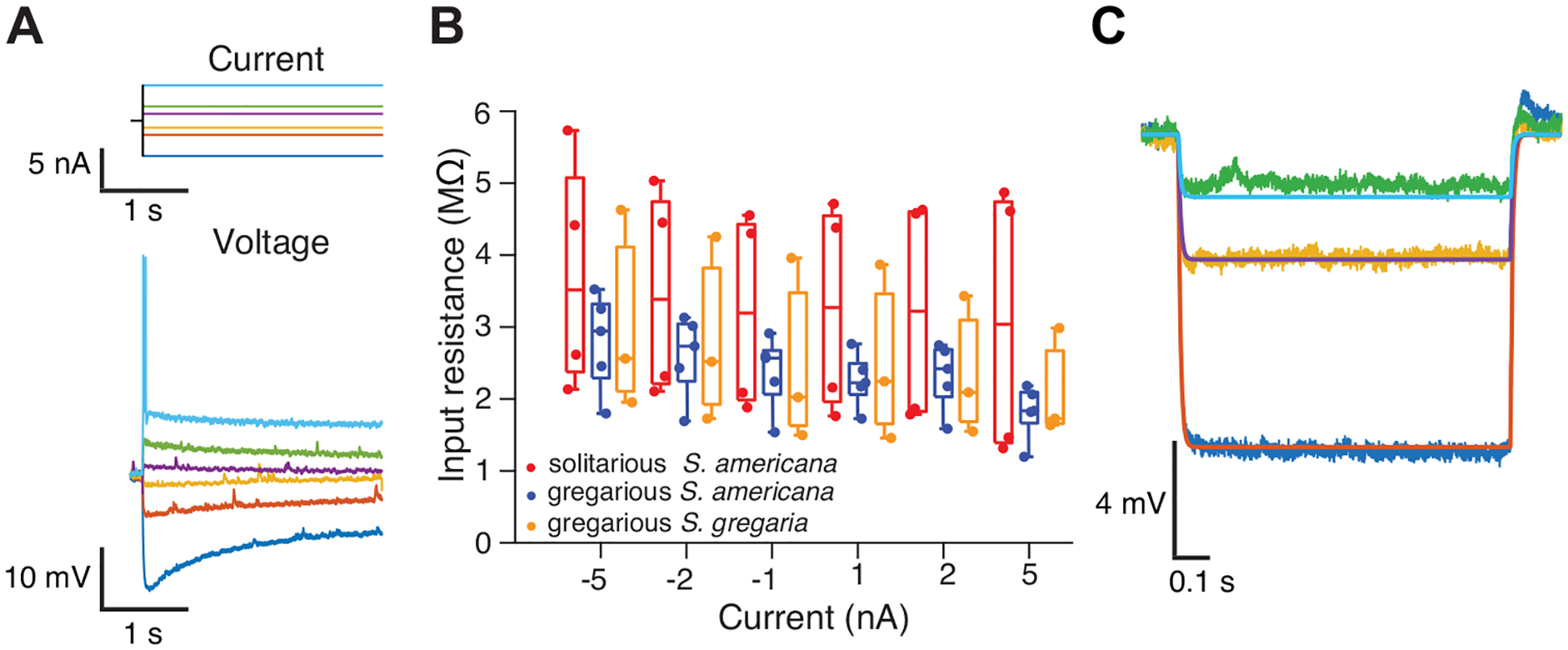
Measuring differences in LGMD membrane input resistance (*R*_i_) to step currents. *A*: recorded *I*_m_ and *V*_m_ traces showing step currents (−5,−2,−1, 1, 2, and 5 nA, *top*) injected into the LGMD and the resulting voltage changes (*bottom*). *B*: comparison of LGMD membrane input resistance in solitarious (*n* = 4), gregarious (*n* = 5) *S. americana* and gregarious *S. gregaria* (*n* = 3). Box plot shows the median, quartiles, and data extents of *R*_i_ values for each group; dots show mean (*R*_i_) value for each animal. None of the *R*_i_ values in the three groups were statistically significant (Kruskal–Wallis test). C: example *V*_m_ traces and best fits of the passive model (injected currents: −1, −2, and −5 nA). *I*_m_, membrane current; LGMD, lobula giant movement detector; *V*_m_, membrane potential.

To test whether the membrane properties in the LGMD differ between the phases, we conducted in vivo dendritic intracellular recordings. For these experiments, we injected both hyperpolarizing and depolarizing current steps into the LGMD and measured the change in membrane potential to calculate input resistance (Fig. 5, A and B). The membrane input resistance (*R*_i_) was measured for four solitarious and five gregarious *S. americana*, and for three gregarious *S. gregaria*. The median *R*_i_ was compared for each of the six input currents between the three groups with no difference at any current (*P* > 0.6, Kruskal–Wallis test; Fig. 5B). The *R*_i_ values for all three groups was within the expected range, although the between animal variability of gregarious *S. americana* was less than found previously (31, 42, 57). Like with the morphology of the neuron (Fig. 4), electrotonic size did not differ between phases.

### LGMD Passive Compartmental Simulations

Comparing membrane properties of the LGMD neuron upon injecting current in silico showed no significant differences in the fitted specific axial resistance (*R*_a_), membrane resistance (*R*_m_), and membrane capacitance (*C*_m_) in three animals from three different groups of grasshoppers. Although the specific axial resistance of the LGMD in gregarious *S. americana* was 72.3 Ω cm, it was 61.2 Ω cm in gregarious *S. gregaria* and 75.0 Ω cm in solitarious *S. gregaria*. The specific membrane resistances were quite close to each other, ranging from 5.9 kΩ cm^2^ in gregarious *S. americana* to 4.6 kΩ cm^2^ and 6.3 kΩ cm^2^ in gregarious and solitarious *S. gregaria*, respectively. The *C*_m_ in gregarious *S. americana* was 1.1 μF/cm^2^, whereas for gregarious and solitarious *S. gregaria* it amounted to 1.4 μF/cm^2^ and 1.1 μF/cm^2^, respectively. These values match with the ones shown in a previous study done on *S. americana* (34). In general, the simulated neuron models accurately fitted the experimental data (Fig. 5C). The average *R*^2^ value for the traces was 0.9.

### Gene Expression in the LGMD Was Consistent between Phases

**Figure 6.**
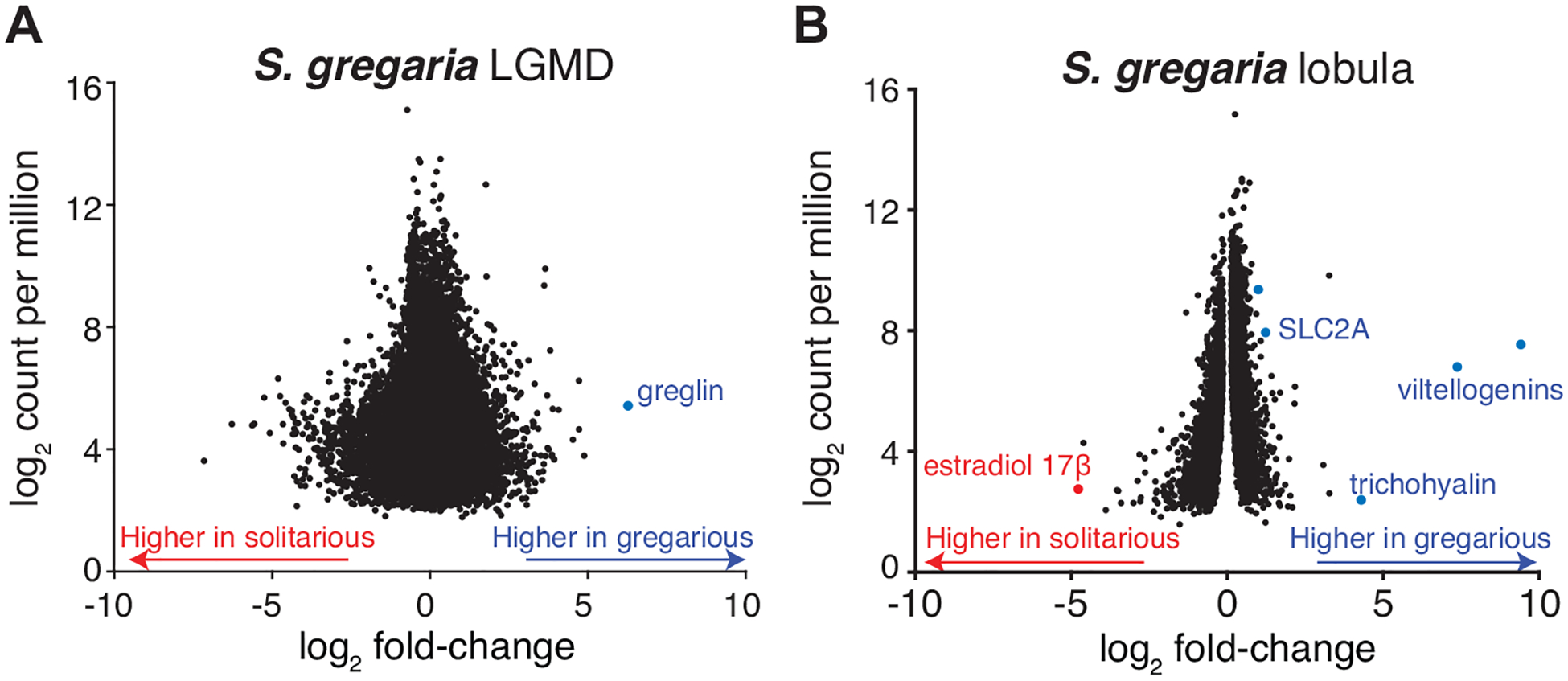
Differential gene expression between two phases of *S. gregaria*. *A*: analysis of single cell RNA-Seq data revealed only one significantly differentially expressed gene (DEG), coding for greglin, within the LGMD. *B*: similarly, among all the genes expressed in the lobula that were analyzed, only six DEGs were found coding for: vitellogenins (LOC126298903 and LOC126298904), trichohyalin (LOC126335968), solute carrier family 2 facilitated glucose transporter (SLC2A; LOC126354871), and an uncharacterized protein (LOC126278381), which were higher in gregarious animals (blue dots), and estradiol 17-beta-dehydrogenase 2-like (LOC126341510) which was higher in solitarious animals (red dot). LGMD, lobula giant movement detector.

Individual LGMD somata were extracted from *S. gregaria* of both phases and the total mRNA of the cells were sequenced (51). In each cell, RNA reads of 13,000–18,000 unique genes were detected. Of these genes, only one gene was differentially expressed between phases, with greglin (LOC126331978) more highly expressed in the LGMD of gregarious *S. gregaria* compared with solitarious animals (Fig. 6A). For each LGMD that was harvested for sequencing, the rest of the lobula was used for a bulk RNA sequencing control. There were only six differentially expressed genes between the phases in the bulk controls. These included a glucose transporter and two vitellogenin genes highly expressed in the lobula of gregarious *S. gregaria* (Fig. 6B). This shows surprisingly little change in gene expression of visual neurons between phases.

## DISCUSSION

We conducted a study wherein we tested for density-dependent differences in looming escape jump responses of *S. piceifrons*, *S. gregaria*, and *S. americana* reared under either solitarious or gregarious conditions. We found that for all three species, the gregarious animals jumped significantly more than their solitarious counterparts (Fig. 2). This study is the first to examine phase differences in looming-evoked escape and the first focusing on a possible phase-dependence in jumping or loom responses in *S. americana* or *S. piceifrons*. As previous studies found the responses of the LGMD to determine looming-evoked behavior, we followed up on these behavioral observations with an examination of the physiology, genetics, and morphology of the LGMD between phases. Surprisingly, we found no differences that could explain the phase-dependence of loom escape jumps.

Phenotypic plasticity results in behavioral, morphological, and genetic changes in animals that are mediated by changes in environmental conditions (35, 58–60). Earlier studies showed that the DCMD firing between the two phases of *S. gregaria* locusts varied significantly (37, 38). The visual system, the motor system, and the musculature have been widely studied in grasshoppers and locusts (37, 38, 61). Until now, not much information was available about the differences in escape behavior, or in electrophysiological and morphological characteristics of the LGMD upon phase change in *S. gregaria* and *S. americana*. This sets the stage for our study and for a side-by-side comparison of the differences in escape behavior, DCMD firing patterns, LGMD shape and size, as well as differential gene expression within the LGMD between phases and species of grasshoppers and locusts. Currently, the only available data for direct comparison with our findings comes from *S. gregaria*.

Indeed, previous studies of *S. gregaria* found that solitarious grasshoppers have more muscular hind legs that help them jump faster and further than gregarious locusts (61). According to Rogers et al. (61), solitarious animals spent twice the energy per jump relative to gregarious ones. This energetic difference could be the reason why our solitarious animals jumped significantly less than gregarious ones. Due to the dearth of alternative explanations for these differences in escape behavior, we suggest that energy conservation in solitarious grasshoppers is a plausible explanation. Besides, we know that in the solitarious stage grasshoppers are cryptically colored helping them blend easily with their surroundings thus increasing their chances of survival by avoiding predation. In contrast, gregarious animals can be brightly colored, and easily visible. This could be another possible reason why solitarious animals are less prone to escape jumping.

Looming escape jumps depend on the firing pattern of the LGMD neuron (28, 31), so we hypothesized that the difference in jump behavior between phases could be an outcome of differences in LGMD activity. To test this hypothesis, we took advantage of the 1:1 firing pattern of the LGMD and its postsynaptic neuron, the DCMD (29). We found that the DCMD firing patterns in the two phases of both species were not significantly different from each other and that they are unlikely to explain the differences in jump behavior (Fig. 3). Similarly, Matheson et al. (38) conducted the first study comparing DCMD firing rates in the two phases of *S. gregaria* and showed that initial responses were identical across phases. Their study showed a clear difference between gregarious and solitarious animals in subsequent trials, presented with short intertrial intervals (but see Fig. 3 of Ref. 37). Thus the main difference in DCMD firing rate between the two phases is due to differences in habituation of DCMD firing rates across phases. Gaten et al. (62) also noticed no differences in response between phases when waiting an hour between looms which would prevent any habituation, in agreement with our findings.

The mechanism behind the previously found differences in habituation across phases remains unclear. Habituation is likely presynaptic to the LGMD based on earlier studies (45) and is mediated by multiple independent neuromodulators, including octopamine and nitric oxide (63, 64). Our RNA-sequencing experiments make it unlikely that the mechanism originates in the lobula since we found no changes either in the LGMD or in the lobula gene expression pattern that could explain habituation changes across phases. This suggests changes in medullary neurons as a possible cause of habituation differences.

Since earlier studies have shown that gregarious *S. gregaria* brains are significantly larger than long-term solitarious ones (35, 36) we compared the morphology of the LGMD between the two phases. There has been no previous comparison of individual cell morphology between phases, though. Our analysis of LGMD dendritic morphology in gregarious *S. americana*, solitarious and gregarious *S. gregaria* showed no significant differences in the dendritic arbors between the two phases of *S. gregaria*. Yet, the dendritic arbor of *S. americana* was smaller than that of *S. gregaria* (Fig. 4).

Having established that there was no difference in the shape or size of the LGMD, we wanted to determine if there were any differences in the membrane properties of the LGMD between the two phases. To answer this question, we recorded intracellularly from *field A* dendrites in vivo and measured membrane input resistance and resting membrane potential. Once again, our data showed that there were no significant differences in the properties of the LGMD between the two phases (Fig. 5). Since neither morphology nor input resistance were different, this suggests that the electrotonic characteristics of the LGMD are also consistent across phases. It is not possible to directly measure the axial resistivity, membrane resistance, or membrane capacitance from these recordings, so we used previously described methods for estimating these properties from current steps using biophysical modeling (34). These simulations involved creating multicompartmental models from the detailed dendritic morphologies of both phases (Fig. 4) and fitting the current clamp data (Fig. 5). The resulting simulations suggested consistent membrane properties across species and phase, with values similar to those found previously for *S. americana* (34).

Previous experiments on the active membrane properties of the LGMD have found specific ion channels to play critical roles in processing of looming stimuli (31, 33). We wanted to see if any receptor or voltage-gated ion channels present in the cell membrane are differentially expressed upon phase change. So, we extracted LGMD somata for single-cell RNA sequencing and found that only greglin was upregulated in gregarious *S. gregaria*. Greglin is a serine protease inhibitor induced by juvenile hormone that is involved in vitellogenesis (egg yolk production in vertebrates and invertebrates) (65, 66). Absence of greglin results in immature oocytes and reduced egg numbers (67). In *S. piceifrons*, RNA sequencing of head tissue found greglin to be upregulated in gregarious locusts (39). The connection between greglin and vitellogenesis is notable as there was increased expression of vitellogenins in the surrounding lobula (Fig. 6B). In addition to its role in egg production, vitellogenin is also involved in immune function and energy metabolism with different expression between castes in the brain of multiple social insect species (68–71). In honey bee brains, the changes in vitellogenin expression were determined to be in glial cells involved in metabolic regulation (71). A glucose-transporter was also upregulated in the lobula of gregarious animals, further suggesting that the gene expression changes found might be due to an increased energy metabolism in gregarious animals.

Gregarious and solitarious locusts differ in coloration, size, and behavior (3). In addition, we found clear differences in escape behavior for both locusts (*S. gregaria* and *S. piceifrons*) and a nonswarming grasshopper (*S. americana*). Despite this we found no differences in morphological, electrophysiological, or genetic properties of the LGMD between the phases that could explain the behavioral difference. This raises the question of where the phase-dependent difference arises and what other neurons might underly the change in behavior. The most likely candidates would be the jump coordination circuitry within the meta-thoracic ganglion. The DCMD synapses directly onto flexor, and extensor motor neurons generating jumps and activity in these neurons differs between phases of *S. gregaria* (27, 37, 61). Phase-dependent neuromodulatory changes of this circuitry depressing the synaptic connection from the DCMD onto these neurons could lead to reduced motor activity.

## Data Availability

All the data is available on the Mendeley database: https://data.mendeley.com/preview/gxrwdk2nvt?a=10c161ef-fd7a-4351-a256-f45cfe145a70; https://doi.org/10.17632/gxrwdk2nvt.1.
